# Exploring beyond norms: social capital of pregnant women in Sri Lanka as a factor influencing health

**DOI:** 10.1186/s40064-016-2063-2

**Published:** 2016-04-06

**Authors:** Thilini Chanchala Agampodi, Suneth Buddhika Agampodi, Nicholas Glozier, Sisira Siribaddana

**Affiliations:** Department of Community Medicine, Faculty of Medicine and Allied Sciences, Rajarata University of Sri Lanka, Saliyapura, Sri Lanka; Brain and Mind Centre, Central Clinical School, Sydney Medical School, University of Sydney, Sydney, Australia; Department of Medicine, Faculty of Medicine and Allied Sciences, Rajarata University of Sri Lanka, Saliyapura, Sri Lanka

**Keywords:** Social capital, Maternal health, Qualitative, Sri Lanka

## Abstract

**Background:**

Social capital during pregnancy in low and middle-income countries is hardly discussed in scientific literature. In Sri Lanka, even though the maternal health indicators are exemplary, addressing social determinants in pregnancy to improve the quality of care remains at minimal levels. While social capital is found to be context dependent, a comprehensive approach on identification of its dimensions within the context will unravel its relationships to health. The present qualitative study protocol was developed to explore social capital related to health among pregnant women in Anuradhapura district Sri Lanka.

**Methods:**

The study will be conducted in two phases. In the phase one, we will select different communities from Anuradhapura district. Five to seven pregnant women will be selected from each community to complete a two week solicited diary on their social relationships. After completion of the diaries they will be interviewed for further clarification of social capital based on their diary documentation. In the second phase, we will conduct in-depth interviews with Public Health Midwives and senior community dwellers from each community to discuss social capital of pregnant women in the respective communities in order to triangulate the information obtained from the diaries. A framework analysis will be conducted for each community and formulate a final framework for social capital among pregnant women and there possible effects on health.

**Discussion:**

This study will focus on filling a research gap of social determinants pertaining to maternal health in Sri Lanka. The findings will be helpful in generating hypotheses on unidentified social risk factors and their pathways to maternal health. The results of this in-depth exploration will be utilized to formulate a culturally sensitive study instrument to assess social capital during pregnancy.

## Background

The World Bank defines social capital as “institutions, relationships and norms that shape up the quality and quantity of a society’s social interactions”. It states that “social capital is not just the sum of institutions in a society, but also the glue that holds them together” (http://go.worldbank.org/K4LUMW43B0). Social capital has two main dimensions; structural and cognitive (Onyx and Bullen 2000). Structural social capital refers to externally observable aspects of social organization (Krishna 2001; McKenzie et al. 2002; Harpham et al. 2002) while cognitive social capital consists of the norms, values, and beliefs of people that influence social participation and mutual support. The most recent approach (Szreter and Woolcock 2004) expresses these same dimensions in three distinct forms, namely “bonding”, “bridging”(horizontal) and “linking”(vertical) social capital. Despite these deliberations the definition and dimensions of “social capital” is still being debated by academics (Portes 1998).

Nevertheless, social capital stands as an area that cannot be neglected in public health service provision, especially in economically poor societies where it could serve as an untapped resource. However in Low and Middle Income Countries (LMIC), it is still a neglected social determinant of health (Agampodi et al. 2015). One reason for this might be lack of applicability of social capital constructs that are currently in use, as this concept was originally developed in HICs. Similarly, one major challenge in the universal application of this concept is its context specificity (De Silva et al. 2006). There is no gold standard tool to measure social capital. While numerous measures are being used, cultural adaptation and validation of these tools are required in different settings to identify context specific factors. Although the number of studies conducted in LMIC is few compared to HICs, our recent systematic review indicates that cognitive social capital in particular is associated with various aspects of health such as mental health conditions, self rated health, child nutrition, adolescent risk behaviors vaccination and road traffic accidents in these regions (Agampodi et al. 2015). However we did not find a proper exploration or analysis of social capital during pregnancy, a key time when aspects of social capital may be vital in maintaining the health of the mother and later, the newborn.

Social determinants of health is on the agenda of global public health during the past decade (Report of Commission of Social Determinants of Health 2008). However, when it comes to maternal care, the global maternal health agenda is still focused on curative care which has not yielded expected results in further reduction of maternal or perinatal mortality in Low and Middle Income Countries (LMIC) (Souza 2013). Even countries like Sri Lanka while being exemplary in maternal care services, has not paid much attention to social aspects of pregnant women in promoting maternal health.

Sri Lanka considers maternal care as a high priority in health service delivery. With a strong public health system and free health services Sri Lanka has achieved 98 % coverage in antenatal care, 96 % institutional deliveries and a maternal mortality ratio of 33.2 per 100,000 live births (Annual Report on Family Health 2013). Observing the evidence based interventions that have lead to this success, we find that identifying the influence of social aspects during pregnancy has minimally addressed in the national maternal care program. Further exploration of literature reveals that there is scarce evidence on social capital in pregnancy even among other LMICs. This paper possesses a protocol for in-depth exploration of social capital in pregnancy, which is a hardly touched (Lamarca et al. 2013) area in public health literature in LMIC.

### Study aim

This study aims to explore and understand contextual differences of social capital of pregnant women in the largest district of Sri Lanka and to identify the pathways of relationship of social capital to health.

## Methods

### Study design

**Fig. 1:**
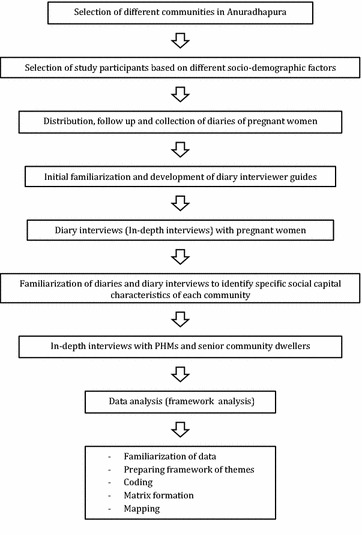
Exploration of social capital of pregnant women in Anuradhapura district; Study flow chart

We will use a qualitative design to explore social capital during pregnancy. The study will consist of two phases (Fig. 1). In the first phase we will explore social capital during pregnancy among pregnant mothers and in the second phase we will triangulate data using grounded theory approach on healthcare providers and community dwellers.

### Study setting and population

The study will be conducted in Anuradhapura, the largest district in Sri Lanka (7179 m^2^). The total population in the district is 886,945, of which 94.1 % is rural (Census on Population and Housing 2012). This is a poor district in Sri Lanka with a median household income around $210 per month (Household Income and Expenditure Survey 2012). The main stay of economy is agriculture. The ethnic breakdown of the population represents as Sinhalese 90.7 %, Tamil 0.8 % and Sri Lankan Moor 8.13 % (Census on Population and Housing 2012). In this district more than 19,000 pregnant mothers are registered annually for antenatal care (Annual Report on Family Health 2013). DHS data shows that antenatal care coverage through public health system is 100 %. Out of the deliveries 98 % take place in health institutions. Access to safe water and sanitation is above 98 % (Monitoring MGDs). According to the DHS, 90 % of females in the district has at least entered secondary level education (Demographic and Health Survey 2007).

### Selection of study sample

#### Phase 1

##### Selecting communities

Under the public health administration framework In Sri Lanka, every district is divided into several Medical Officer of Health (MOH) areas. A doctor with additional training in public health is the chief administrative officer in a MOH area. There are no documented data on culturally and socioeconomically different communities in the district. Hence, we used MOH and other key informants to identify different communities. Each MOH (24 in the whole district) was contacted and interviewed about different communities in their area. This enabled us to identify five different communities in the district. These include; urban or semi-urban, rural, new (Mahaweli) settlers, ancient villages and minority ethnic groups. The new settlers came from other parts of the country and settled in the district under the major irrigation project (Mahaweli) in 1980s. The ancient villagers represent the ancestors of Sri Lanka the “Yaksha gothra”. In addition to these “generally” known communities, we selected three more communities purposefully from the rural sector based on our experience in field service provision. One to represent the recent North-East conflict affected population, situated in the northern border of the district (Padaviya), one to represent a less educated, comparatively resource poor community in which people believe that the health related behaviors are different (Vilachchiya) and another to represent the population which might have influence of ancient Buddhist culture (Mihinthale; the place which Buddhism was first introduce to Sri Lanka).

Communities selected

- *Urban or semi*-*urban community*– Nuwaragam Palatha East
- *Rural*—Nuwaragam Palatha Central (NPC),
- *Other rural communities*
- Community with ancient Buddhist culture—Mihinthale
- Resource poor community—Vilachchiya
- Conflict affected community—Padaviya
- Mahaweli resettlers—Rajanganaya
- Ancient villagers—Medawachchiya
- Minority ethnic communities—Gambirigaswew

##### Selecting study participants

A network of public health workers provides services in MOH areas. These include supervisory officers such as Public Health Nursing Sisters (PHNSs) and supervising public health midwives (SPHMs) as well as grass root level public health workers namely PHMs and Public Health Inspectors (PHIs). Each MOH area is divided into several PHM areas. The PHM is the grass root level health care provider for women and children. A PHM caters for a population of 3000–5000 and resides in her area. She provides domiciliary as well as clinic care for pregnant mothers and children less than 5 years of age. Each and every woman who is eligible to be pregnant is registered under the PHM. Therefore she starts providing services to women before they become pregnant (pre-conceptional care) and continue and follow them up until their children complete 5 years of age. She participates in risk screening, health education and care provision of pregnant mothers as well as provision of growth monitoring, promotion of infant feeding, immunization and Early Childhood Care and Development for children (Development of Reproductive Health Services 2009). Due to this reason she is always in contact with the women in the community and has a good reputation among villages. During field care she visits around 14 households per day. We will select study participants for phase 1 with the help of the PHM of the specific community. Five to seven pregnant women in different gestational periods, having different educational levels and socio-economic backgrounds will be selected to represent each community with the PHMs help. Initially five from each area will be selected and additional participants will be selected up to the point of data saturation.

#### Phase 2

One PHM and a community dweller domiciled in that area for a long period, and able to describe about social relationships will be selected as key informants for this study.

### Study tools

#### Phase 1

##### Participant diaries

Participant diaries have been identified as a valid source of data in qualitative health research (Jacelon and Imperio 2005). They have been previously used in pregnancy as a source of acquiring data on physical activity (Evenson et al. 2012). In solicited diaries, the study participants complete the diary reflecting on the issues requested by the researcher (Elliott 1997). Diary data is meant to have high social validity. The advantage of diary method lies in the concurrent reporting of participant behaviors in a natural setting which is otherwise not available for the researcher (Follick et al. 1984). Use of diaries reduces the recall bias and telescoping effects that occur in retrospective interviews but depend on the writing literacy in the sample (Furness and Garrud 2010). This method, combined with participant interviews, can be used as an approximate substitute to the participant observation (Zimmerman and Wieder 1977). Participant observation is one of the best methods of exploring social capital inside their sociocultural milieu (“emic” paradigm) (Sumathipala and Murray 2000). Through solicited diaries we will encourage participants to document daily social relationships and their reflections and underlying norms and also asked them to express how they feel that these relationships might influence their health.

The diary will be prepared according to the common guidelines (Corti 1993). A half A4 30 page booklet will be used as a diary. An information sheet on the purpose of the diary, a list of open ended questions/statements that will guide the participant to document (Table 1) and a model sample page of a already written diary was included at the beginning of this booklet. The pages will be paginated according to the calendar days of the week. Two pages will be provided for a single day. At the end a page is provided to document participant’s own comments or clarifications.

**Table 1:** The open-ended questions included in the diary as a guide for documenting

At the end of each day,
Can you describe the relationships you had and activities performed together today with the members of your household?
How did the people in your household help you?
What do you think about these relationships?
How did you feel today regarding the relationships of the members within your household?
Did you meet, communicate or worked together with any other person outside your household today? If so can you describe about this event?
How did you feel about these relationships?
How do you think these relationships might influence your health?

For the participants that are not literate we would offer an audiotape diary. For this a tape recorder, two 60-min cassette tapes and a demonstration of how to use the machine will be provided. A separate recording on purpose of the diary and above mentioned guidelines including a model recording will also added. For participants who are less technologically minded a family member will be trained for facilitate the recording.

#### Phase 2

The study tool of the *phase 2* is an in-depth interviewer guide. It will be prepared to include the views of key informants on the community about the social capital of pregnant women. The questions in these guides will be based on the initial diary entries to achieve more clarification of social capital of pregnant women in each community (Table 2).

**Table 2:** In-depth interviewer guides

Themes
Community characteristics
Boundaries of the relationship network of a pregnant woman in the area
Relationships of pregnant women within household, neighbors, friends and relatives
Observation of sense of belonging, social isolation and social contribution of a pregnant women
Social participation of a pregnant women Health seeking behavior and access to health resources How common problems in the community affects pregnant women

### Training of interviewers

Pre-intern medical graduates, who are trained on qualitative methods and interviewing skills will conduct interviews. An investigator who possessed expertise on qualitative techniques will further explain about the procedures through mock interviews.

### Piloting

The study tools will be pilot tested in one community. Piloting will be conducted to test the study tools and to assess and improve the interviewing skills of interviewers. We will assess whether the pregnant women will understand what is expected from them when engaged in diary writing. Practical issues such as whether the space provided in the diaries is adequate will be assessed during the piloting. The interviewer guides will be tested to see whether the participants understand the cognitive interpretations of the questions.

### Data collection

#### Diaries and diary interviews

Data collection will be carried out in a one community at a time. The selected participants will be briefed orally and with a participation information leaflet and written informed consent will be obtained after an interval. The participants will be instructed to document for 2 weeks, which is the optimum duration for solicited diaries (Jacelon and Imperio 2005). After distribution of diaries we will contact the participants through mobile phones 2–3 times during the period of writing, to remind them and to clarify their doubts about the documentation. After collection of diaries, the investigators will read and identify entries related to social capital that needs further clarification. An interviewer guide is prepared by TCA for each participant to obtain the clarifications needed. Hence, there will be a separate diary interviewer guide for each participant. The diary interviews will be conducted within 1 month after completion of diaries.

#### In-depth interviews

In-depth interviews will be conducted at a place convenient for the key informants. Probably at field maternal clinics and some at community dwellers residence. We will use Family Health International (FHI) guidelines to conduct the interviews (Mack et al. 2005). Interviews will be tape-recorded and notes will be taken with the consent of the participant. If new concepts arise during the study The investigators would go back and interview the previous interviewees on these concepts where ever necessary.

### Data expansion, transcription and storage

We will expand the data immediately after each interview, and transcribe within 1 month and store in soft and hardcopy file for each community. An investigator fluent in both English and the mother tongue of the community will translate the diaries and the interview notes of the minority ethnic community. Only the investigators will have access to data.

### Planned analysis

We will conduct framework analysis (Pope et al. 2006). Framework analysis is a deductive method of qualitative analysis (others; grounded theory and thematic analysis) used to analyze diaries (Corti 1993). It is used in studies where the intended outcome is typically set in advance and shaped by the information that is gathered (Pope et al. 2006). Two investigators will initially familiarize themselves by reading and re-reading the notes and transcripts. Secondly thematic framework will be outlined, in a hierarchical basis to form main themes and sub themes that will include dimensions and constructs of social capital in pregnant women in different communities. These headings will be used to index original data. The initial thematic framework and indexes will be refined during further analysis and this developmental work will be recorded. The original data that are indexed will be summarized and charted under the identified themes to form a matrix format, initially for each community and then for the whole district. The range and nature of social capital will be mapped using bubble diagrams to describe conceptual dimensions. Participants’ expressions will be used to formulate pathways between social capital and health. The process will be conducted manually as data management software is not available in native language.

### Research rigor and quality control

Following steps will be taken to ensure quality and trustworthiness of this study.

#### Streamlining data collection methods, tools and analysis

All data collection methods and tools will be designed according to currently accepted guidelines (Corti 1993; Mack et al. 2005). The diaries, interviewer guides and note-taker forms will be prepared early and pretested and checklists will be used in all field visits as described by the FHI guidelines (Mack et al. 2005) to ensure quality of data collection. Interviewers were trained and supervised throughout the study. Data analysis methods will be done paying attention to rigor and transparency of the procedures. Two investigators trained in qualitative analysis and knowledgeable on the concept of social capital will participate in data analysis. The investigators will collectively perform the framework analysis simultaneously to improve the reliability in indexing and mapping. When different opinions arise, these will be discussed among the investigators until consensus.

#### Triangulation

Collecting information from a diverse range of individuals using variety of methods minimizes bias due to chance and systematic biases (Maxwell 2013). We use three different techniques—diaries, diary interviews and in-depth interviews—and three different types of individuals—pregnant women, PHMs and senior community dwellers to gather knowledge on social capital of pregnant women in each community.

#### Respondent validation

In diary interviews and in-depth interviews we will ask participants whether they would agree with social capital constructs that we have interpreted from the diaries.

#### Reflexivity (Maxwell 2013)

One anticipated bias was whether the participant pregnant women would change their personal relations when documenting on social relationships at daily basis. To overcome this participants were informed prior and were asked to avoid adopting behaviors other than natural in their day to day lives. Also they were assured that their relationships would not be judged and commented but only probed. Other aspect was in interpretation of data in specific communities by the investigators. We were doubtful whether our prior knowledge on these different communities would interfere when conducting interviews and in documentation. To address this investigator maintained diaries with memos throughout the study with the expectation of using them in interpretation and discussion of our findings.

### Ethical considerations

This study is based on participant diaries and interviews. Although information on social capital does not generally involve sensitive issues, reflection of ideas especially in diaries may sometimes create emotionally sensitive situations for the participants. As the investigators will follow these participants throughout the duration of diary writing, mothers that faced such situation will be allowed to discontinue if they wish to and will be referred to psychological assessment if necessary.

Possible invasion of privacy will be minimized by de-identifying participants and replacing names. Confidentiality will be maintained throughout the data collection analysis and presentation.

Ethical clearance will be obtained from the Ethics and Research Committee, Faculty of Medicine and Allied sciences, Rajarata University of Sri Lanka.

## Discussion

The present study will focus on one of the neglected social determinant in maternal care service provision. It will provide an in-depth exploration of social capital among pregnant women in the given area and would guide us to identify the pathways as to how it could influence health. Non-effective penetration of centrally mediated health programs at the peripheral level has been a common problem in public health service provision. This study would be beneficial in identifying the community characteristics essential to implement health promotion strategies such as positive deviance, mother support groups and involving family members in improving maternal wellbeing. The study will also reflect the importance of studying these characteristics before laying down policies at central level in a country.

The results of this study will be used to formulate a study instrument to measure social capital among pregnant women. To date we have not observed a specific social capital tool to be used in pregnancy despite the observation that social networks, relationships and there depth of a pregnant woman might readily differ from a normal individual in a population.

To date self-written diaries are hardly used to explore social capital in LMICs (Agampodi et al. 2015). The diary and the diary-based interview will provide the investigator and opportunity to view individual social capital as well as community/neighborhood social capital. The ultimate aim of this explorative study is to generate hypotheses on social capital as a risk/protective factor for pregnant women. This will be specific to the community. This study will act as a basis for generation of research on maternal social capital in LMICs and to rethink whether social capital should be incorporated into the routine risk assessment in pregnancy.

## Conclusion

This study protocol will include transparent systematic qualitative methodology to explore and capture social capital of pregnant women and its implications to health in a given context.
